# Comparison between measured and calculated dynamic wedge dose distributions using the anisotropic analytic algorithm and pencil‐beam convolution

**DOI:** 10.1120/jacmp.v8i1.2370

**Published:** 2007-02-28

**Authors:** Paola Caprile, Carlos Daniel Venencia, Pelayo Besa

**Affiliations:** ^1^ Pontificia Universidad Católica de Chile Centro de Cáncer “Nuestra Señora de la Esperanza,” Santiago Chile

**Keywords:** dynamic wedge, dose distribution comparison, calculation algorithms, AAA, PBC

## Abstract

We used the two available calculation algorithms of the Varian Eclipse 7.3 three‐dimensional (3D) treatment planning system (TPS), the anisotropic analytic algorithm (AAA) and pencil‐beam convolution (PBC), to compare measured and calculated two‐dimensional enhanced dynamic wedge (2D EDW) dose distributions, plus implementation of the dynamic wedge into the TPS. Measurements were carried out for a 6‐MV photon beam produced with a Clinac 2300C/D linear accelerator equipped with EDW, using ionization chambers for beam axis measurements and films for dose distributions. Using both algorithms, the calculations were performed by the TPS for symmetric square fields in a perpendicular configuration. Accuracy of the TPS was evaluated using a gamma index, allowing 3% dose variation and 3 mm distance to agreement (DTA) as the individual acceptance criteria. Beam axis wedge factors and percentage depth dose calculation were within 1% deviation between calculated and measured values. In the non‐wedged direction, profiles exhibit variations lower than 2% of dose or 2 mm DTA. In the wedge direction, both algorithms reproduced the measured profiles within the acceptance criteria up to 30 degrees EDW. With larger wedge angles, the difference increased to 3%. The gamma distribution showed that, for field sizes of 10×10 cm or larger, using an EDW of 45 or 60 degrees, the field corners and the high‐dose region of the distribution are not well modeled by PBC. For a 20×20 cm field, using a 60‐degree EDW and PBC for calculation, the percentage of pixels that do not reach the acceptance criteria is 28.5%; but, using the AAA for the same conditions, this percentage is only 0.48% of the total distribution. Therefore, PBC is not reliable for planning a treatment when using a 60‐degree EDW for large field sizes. In all the cases, AAA models wedged dose distributions more accurately than PBC did.

PACS numbers: 87.53.Bn, 87.53.Dq, 87.53.Kn

## I. INTRODUCTION

Wedge‐shaped isodoses are required in many clinical situations. Sloping distributions can be obtained by inserting a physical wedge in the beam. However, physical wedges have several unfavorable functional and dosimetric characteristics^(1)^ such as beam hardening, fixed wedge angles, and limited field size.

With the advent of computer control, the simulation of wedge filters by movement of the collimator jaw during the irradiation process becomes possible.^(2)^ The dynamic wedge (DW) has considerable advantages over the physical wedge. The use of DW requires accurate configuration of the treatment planning system (TPS). Implementation of DW requires measurement of percentage depth doses (PDDs), beam profiles, and wedge factors (WFs). Measurements must take into account the need for integration of the dose at the measurement point for the wedged fields during the entire exposure. The procedure has been described in previous works.^(^ ^3^ ^–^ ^6^ ^)^

Varian Enhanced Dynamic Wedge (EDW—Varian Medical Systems, Palo Alto, CA) provides seven wedge angles (10, 15, 20, 25, 30, 45, and 60 degrees), generating sloping dose distributions by moving one of the jaws with variable speed while the opposite remains steady. The relationship between jaw position and the percentage of total monitor units delivered is described in a segmented treatment table. The segmented treatment table is used to generate the primary intensity function matrix used by the pencil‐beam convolution (PBC).^(7)^

The Varian Eclipse TPS (Varian Medical Systems) employs two calculation algorithms, the anisotropic analytic algorithm (AAA) and a PBC. Even though the PBC does not consider extrafocal radiation, it agrees well with the measurements in the field axis; however, it can present inaccuracies in the calculation of dose distributions at the field corners, especially for rectangular‐ and square‐wedged fields.^(8)^ The AAA uses pre‐calculated Monte Carlo simulations based on a convolution model^(9)^ and takes into account extrafocal radiation and electron contamination.^(10)^

Several works that compare measured and calculated dose in the field axis using DW, such as Papatheodorou et al. 1998,^(3)^ Bayouth and Steinberg 1997,^(5)^ and Samuelsson et al. 1997^(7)^, conclude that the TPS accurately models the wedged dose distributions for symmetric fields, with dose variations below 2% of the normalization or 2 mm for regions with high dose gradient. However, no reports using DW compare measured with calculated two‐dimensional (2D) dose distributions.

The present work compares EDW between measured 2D dose distributions in the plane perpendicular to the beam axis and those generated by the TPS using the PBC and the AAA.

## II. MATERIALS AND METHODS

A linear accelerator, Clinac 2300 C/D (Varian Medical Systems), was used to produce a 6‐MV photon beam and generate the EDW. The dose distributions were calculated by the three‐dimensional (3D) Eclipse 7.3 TPS using the AAA and the PBC. Measurements were carried out for square fields, with a 100‐cm source‐to‐surface distance (SSD) configuration, for seven EDW angles and open field.

### A. Calculation algorithms

#### A.1 Pencil‐beam convolution

The PBC model generates the dose distribution matrix by convolution of pencil‐beam kernels with a non‐uniform field function; the pencil‐beam kernels are derived from open‐beam measurements.^(11)^

The DW dose calculation^(12)^ uses only open‐field data. Dose distribution is determined by superposition of asymmetric rectangular fields in the wedge direction. The contribution of each asymmetric field is calculated convolving the scatter kernel with the field function, which takes a value of 0 or 1 depending on whether it is considered to be inside or outside each field. Depth dose is obtained by the addition of all asymmetric field contributions.

#### A.2 Anisotropic analytic algorithm

The AAA is a 3D pencil‐beam convolution superposition algorithm that has separate modeling for primary photons, scattered extrafocal photons, and electrons scattered from the beam‐limiting devices.

Dose calculation is based on separate convolution models for primary and extrafocal photons, contaminating electrons, and photons scattered from the beam‐limiting devices. Clinical broad beam is divided into small, finite‐sized “beamlets” to which the convolutions are applied. The final open‐field dose distribution is obtained by superposition of the dose contribution calculated with the photon and electron convolutions for the individual beamlets.^(9)^

The DW dose calculation is computed by using the PBC algorithm plus superposition of asymmetric rectangular fields in the wedge direction.

### B. Commissioning

The AAA and PBC require the same set of dosimetric data for commissioning. The TG‐53 protocol from the American Association of Physicists in Medicine was used to acquire experimental data for commissioning. The measurements performed included output factors for fields with dimensions 2, 3, 4, 6, 8, 10, 12, 15, 20, 25, 30, 35, and 40 cm (square fields and combinations of these) and dose profiles at $d_{\max}$ (15 mm) and 50, 100, 200, and 300 mm. Relative dosimetry was performed using a PTW PinPoint 31014 ionization chamber (PTW Freiburg, Freiburg, Germany) and a Blue Phantom (Scanditronix Wellhofer, Uppsala, Sweden). For PDD measurements, we used a Wellhofer IC‐10 (Scanditronix Wellhofer) cylindrical ionization chamber in addition to the PinPoint.

### C. Evaluation of calculations

We evaluated the TPS by comparing measurements and calculations performed under the same conditions, based on a phantom created by the TPS with a homogeneous density of 1 $g/{\text{cm}}^{3}$, emulating a plastic water phantom with density of 1.013 $g/{\text{cm}}^{3}$. The slice spacing and resolution of the phantom were 1 mm and 0.78×0.78 mm respectively. The calculation grid was 2.5×2.5 mm for the PBC and AAA calculations.

#### C.1 Beam axis

Measurements were performed in a water phantom MT‐DDA (Med‐Tec, Orange City, IA) with a 0.125‐cc Semiflex 31010 ionization chamber (PTW Freiburg), and a Keithley MK614 electrometer (Keithley Instruments, Cleveland, OH).

Measurements of WF were performed for two depths (5 and 10 cm) and for square fields of dimensions 4, 5, 6, 8, 10, 12, 15, and 20 cm. The PDDs were determined for 10×10 cm and 20×20 cm fields at depths of 1.5, 5, 10, 15, 20, 25, and 26.7 cm.

#### C.2 Off‐axis

Dose distributions were measured using EDR2 Ready‐Pack films (Eastman Kodak Company, Rochester, NY). Film irradiation was performed at 5 cm depth in a plastic water phantom for 4×4, 10×10, 15×15, and 20×20 cm field size settings. To reduce the variability of working conditions, the calibration^(13)^ and dosimetry measurements were performed in a single session.

An automatic film developer (FPM 2800: FUJI Photo Film, Tokyo, Japan) was used. The films were scanned using a Vidar VXR‐12 film scanner (Vidar Systems Corporation, Herndon, VA) for later analysis with RIT 113 V4 software (Radiological Imaging Technology, Colorado Springs, CO).

Measured dose distributions and the TPS‐generated dose matrix were imported by RIT for dose distribution comparison (profiles and 2D distributions). The criterion employed to evaluate the accuracy of the TPS calculations was the gamma index,^(14)^ with individual acceptance criteria of 3% dose difference and 3 mm distance to agreement (DTA).

## III. RESULTS

During the commissioning, it was possible to verify for the open fields a better adjustment of the penumbra region when using the AAA than when using the PBC.

The variation was less than ±1% between the WFs measured at 5 and 10 cm depth. These factors decrease with the increase of wedge angle and field size in the wedged direction. Calculations made with the available algorithms adjust to the beam axis measurements with less than 1% variation.

The PDD measurements for the seven wedge angles exhibited a maximum deviation of ±0.9% with respect to the open‐field PDD. The difference between measured and calculated PDDs remained under ±1% for both algorithms (comparing either open or wedged fields).

**Figure 1:**
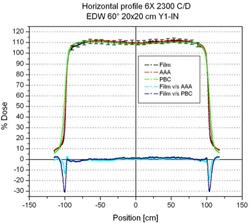
Measured and calculated horizontal (non‐wedged direction) profiles for a 60‐degree enhanced dynamic wedge (EDW) 20×20‐cm field. Cyan and blue lines represent the difference between measured and calculated profiles using the anisotropic analytic algorithm (AAA) and pencil‐beam convolution (PBC) respectively. Error bars represent 2% of the normalization dose.

The calculated profiles in the non‐wedged direction for all measured fields exhibited variations of less than 2% of the dose at the normalization point (central axis) or 2 mm in large dose gradient regions. These results were obtained using the AAA and the PBC (Fig. 1).

**Figure 2:**
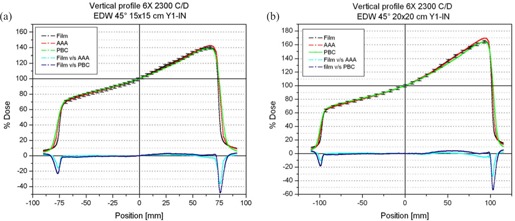
Measured and calculated wedge profiles for a 45‐degree enhanced dynamic wedge (EDW). Cyan and blue lines represent the difference between measured and calculated profiles, using the anisotropic analytic algorithm (AAA) and pencil‐beam convolution (PBC) respectively. (A) 15×15‐cm field. (B) 20×20‐cm field. Error bars represent 2% of the normalization dose.

**Figure 3:**
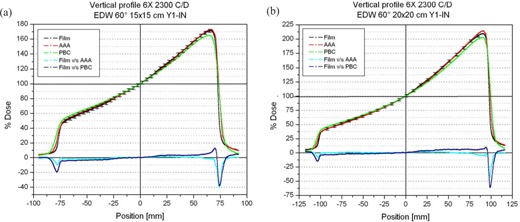
Measured and calculated wedge profiles for a 60‐degree enhanced dynamic wedge (EDW). Cyan and blue lines represent the difference between measured and calculated profiles, using the anisotropic analytic algorithm (AAA) and pencil‐beam convolution (PBC) respectively. (A) 15×15‐cm field. (B) 20×20‐cm field. Error bars represent 2% of the normalization dose.

In the wedged direction, the penumbra region was observed to be well modeled by the AAA (Fig. 2). For wedge angles of 45 and 60 degrees, the PBC always underestimated the maximum dose and slightly overestimated the dose in the “heel” region (Figs. 2 and 3).

Both algorithms reproduced the measured profiles with deviations below 2% of the normalization dose for wedge angles up to 30 degrees EDW. For larger wedge angles using large field sizes, especially in the “toe” region, the difference for the profiles can increase to more than 3% for the PBC calculations (Fig. 3). In every case, the AAA modeled the beam profiles more accurately.

**Figure 4:**
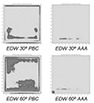
Gamma distribution for a 20×20‐cm field using the anisotropic analytic algorithm (AAA, left panels) and pencil‐beam convolution (PBC, right panels), for 30 degrees (upper panels) and 60 degrees (lower panels) enhanced dynamic wedge (EDW). The upper parts of the distributions represent the low‐dose region of the wedged field; green indicates regions where gamma≤1; and red indicates gamma > 1 (individual criteria: Δ%D=3%, DTA=3 mm).

Fig. 4 shows gamma distributions for different wedge angles; the measured dose distributions were compared with those calculated using the AAA and the PBC. It is important to note that the region of the distribution in which the calculation fails grows with the wedge angle. It is also evident that the penumbra is consistently well modeled by the AAA. However, the most important difference between the two algorithms can be observed in the field corners and the high‐dose region, where the difference between measured and calculated dose distributions reaches 7% or more. The difference between the methods increases for larger wedge angles and larger field sizes.

**Figure 5:**
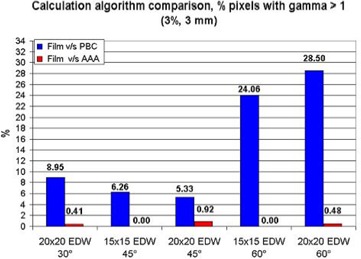
Percentage of pixels in the dose distribution comparison with gamma > 1 and individual acceptance criteria of ΔD=3% and DTA=3 mm, considering the penumbral zone. Blue bars correspond to the film–pencil‐beam convolution (PBC) comparisons, and red bars to the film–anisotropic analytic algorithm (AAA) comparisons.

A quantitative analysis of the dose distribution comparison confirms the differences indicated previously. The plot (Fig. 5) shows the percentage of pixels in the distribution that exceed the acceptance criteria for both calculation algorithms. In all cases, it is easy to note that the AAA models the dose distribution more accurately than the PBC does. Both algorithms from the TPS adequately model the wedged distribution dose up to 45 degrees, with a small percentage of pixels beyond the acceptance criterion for the gamma index: maximum of 5.33% of the distribution for a 20×20 cm field with 45 degrees EDW. The disagreement regions correspond almost completely to the field edges, where the penumbra is not well modeled. For large field sizes with 60 degrees EDW, the percentage of pixels beyond the acceptance criterion is less than 1% using the AAA, but can increase up to 28.5% of the distribution using PBC.

## IV. DISCUSSION

Consistent with previously published data, PDDs and WFs show good agreement with the TPS calculations. Measurements of PDD reveal that the inclusion of EDW into the treatment does not produce beam hardening.

Off‐axis measurements demonstrate additional differences with the calculations from both algorithms. Measured beam profiles in the wedge direction do not exhibit greater deviation from those calculated, except for the penumbra region with the PBC. The difference for the PBC is related to the fact that pencil‐beam kernels, used to generate the dose distribution matrix, require open‐field data measured with an ionization chamber (in this case). The chamber integrates the dose within its cavity, resulting in a less sharp penumbra (high dose gradient region) than the one measured with film, which has a spatial resolution limited only by the resolution of the scanner. It is then possible to think that the width of the penumbra is more related to the measurement method than to the algorithm itself.

Analysis of the entire dose distribution for PBC demonstrates that, apart from the difference in the penumbra region, when the field size and wedge angles are increased, the high‐dose region and field corners of the calculated distribution do not fulfill the acceptance criteria (maximum of 3% dose difference or 3 mm DTA deviation). The variation can be explained by taking into account the fact that the PBC calculates the wedged distribution using a superposition of many rectangular fields without considering extrafocal radiation. The fact that many open fields are superposed to model the DW, considering that inaccuracies are a consequence of the open field modeling, yields greater inaccuracies for this kind of treatment.

On the other hand, the AAA calculates the open‐field distribution considering primary and extrafocal radiation from head‐scatter effects. Therefore, because the open‐field modeling is better with the AAA, wedged dose distributions calculated with this algorithm (by the same method as the PBC) are closer to measured ones.

Using both algorithms, the TPS models the dose distribution accurately up to 45 degrees EDW, with more than 90% of the dose distribution having a gamma index below 1. However, for large fields with 60 degrees EDW, only the AAA models the dose distribution within the established tolerance, achieving the acceptance criteria in more than 99% of the distribution for all the measured field sizes using the available wedge angles. For 60 degrees EDW and large fields, the PBC does not accurately model the dose distributions.

## V. SUMMARY AND CONCLUSIONS

Dynamic wedges have many advantages as compared with physical wedges, such as no beam hardening and lower peripheral doses, but to be used in clinical treatments, they require a complete implementation. “Complete implementation” means that, besides beam axis measurements (PDDs and WFs) and beam profiles, evaluation of the calculated dose distributions is important, especially for large field sizes when using 60 degrees EDW.

The present work shows that, for all studied conditions, the AAA models wedged dose distributions more accurately than the PBC does; the difference between the algorithms are more significant for large wedge angles and large field sizes. It must be emphasized that the use of PBC for planning large‐field treatments with 60 degrees EDW could lead to inaccuracies of clinical significance.

Future studies should evaluate TPS precision in modeling dose distributions for asymmetric fields and parallel opposed configurations.
